# Editorial: Microbiome in an immunocompromised host- a jungle of challenges or a glacier of hidden opportunities?

**DOI:** 10.3389/fcimb.2025.1569842

**Published:** 2025-04-23

**Authors:** Bartosz Wojciuk, Emilia Liana Falcone, Roland Lawson

**Affiliations:** ^1^ Department of Diagnostic Immunology, Faculty of Medicine, Pomeranian Medical University in Szczecin, Szczecin, Poland; ^2^ Department of Medicine, University of Montreal, Montreal, QC, Canada; ^3^ Center for Immunity, Inflammation and Infectious Diseases, Montreal Clinical Research Institute, Montreal, QC, Canada; ^4^ Department of Microbiology, Infectious Diseases, and Immunology, University of Montreal, Montreal, QC, Canada; ^5^ University of Limoges, Inserm U1248, Pharmacology & Transplantation, Limoges, Nouvelle-Aquitaine, France

**Keywords:** immunocompromised host, microbiome, mNGS, inborn error of immunity, secondary immunodeficiencies

It has been over 20 years since Pirofski and Casadevall distinguished the terms “infection,” “colonization,” and “commensalism” by establishing the “damage response framework” in which microbial pathogenesis is the outcome of an interaction between host and microorganism. Accordingly, the resulting amount of damage to the host, as a function of time, can be used to define and characterize the outcome of infection as the respective states of commensalism, colonization, latency or disease (Casadevall and Pirofski, 2003). This concept appears to be especially relevant in the era of multi-omic analysis and the increasing number of patients diagnosed with inborn errors of immunity (IEIs) or affected by secondary, including iatrogenic, immunodeficiency.

It is widely accepted that the microbiome plays a critical role in the maintenance of immune homeostasis, but it can also be an important driver of immune dysregulation, predisposing individuals to infections and metabolic disorders. Although relatively unexplored, the role of the microbiome in the context of impaired immunity has been garnering significant interest. Our objective is to highlight the potential implications of host-microbiome interaction profiles in the context of immune deficiency and multi-omic technology. We take this a step further by describing the practical diagnostic and therapeutic potential of using a metagenomic next-generation sequencing (mNGS) approach in the immunocompromised host. We therefore present a diverse and synergistic collection of studies on the composition and function of the microbiome from different sites in immunocompromised hosts comprising three original papers, two reviews and one case study. To compare the impact of acquired versus inherited immunodeficiency on the composition and function of the microbiome, we included metagenomic studies in patients with IEIs and secondary immunodeficiencies.

Gastrointestinal inflammatory conditions are frequent in patients with IEIs, and this is especially true in common variable immunodeficiency (CVID), one of the most prevalent IEIs. Previous studies have highlighted the important role of the gut microbiome in CVID. This is likely to be influenced by the intestinal microbiota, which, in turn, is influenced by mucosal alterations induced by the underlying immune defect itself. Although fecal microbiota transplantation (FMT) has been considered as a therapeutic option in CVID, there have been concerns about increased intestinal barrier permeability and microbial translocation in these patients (Varricchi et al., 2021; Poto et al., 2023). To date, microbiome-modifying interventions have only been reported in animal models (Hajjar et al., 2023). Among the studies included in our Research Topic, Napiórkowska-Baran et al. present, to our knowledge, the first reported case of successful FMT in a patient with CVID and secondary immunodeficiency post-splenectomy. As suggested by the authors, intestinal dysbiosis underlies gastrointestinal disorders associated with CVID, which are likely resistant to immunoglobulin supplementation and can be successfully treated with FMT.

Next, we explore the use of mNGS in clinical diagnostics and highlight the possible advantages of promoting a hypothesis-free microbiome-focused approach in the clinical setting. This is especially true when considering bacterial pathogenicity as a continuum dependent on host immunity, as outlined in the “damage response framework”. In our Research Topic, Ye et al. present the outcomes of a metagenomic diagnostic investigation in renal allograft recipients. The authors indicate the simultaneous presence of identical microorganisms at different anatomical sites while recognizing complex microbial profiles at specific locations. They also highlight molds such as *Mucor* and *Aspergillus* spp. as clinically relevant infectious agents, although less prevalent in the context of kidney transplantation. Accordingly, other contributors review the potential application of metagenomics in patients with hematological diseases at high risk of febrile neutropenia (Wang et al.) and in patients with hematological conditions both with and without neutropenia (Chen et al.). As suggested by Wang et al., identifying the causal pathogen during febrile neutropenia remains challenging when using traditional microbiological diagnostic methods, while mNGS enables the proper identification of the spectrum of microorganisms coexisting in a patient as it relates to their clinical characteristics and outcomes. Similar to Ye et al., the authors draw attention to less-considered but clinically significant fungi and viruses. Similarly, Chen et al. present the outcomes of a metagenomic diagnostic investigation in the setting of hematological diseases and their complications. In line with the other two studies, the authors indicate the usefulness of mNGS in this specific clinical context and emphasize its ability to identify various microorganisms within one anatomical site.

Previous studies have largely investigated the use of metagenomics as a diagnostic tool in particularly challenging clinical conditions such as central nervous system diseases where it can be used to identify a spectrum of viruses and bacteria in cerebrospinal fluid (Piantadosi et al., 2021; Castellot et al., 2023). The diagnostic application of metagenomics in immunodeficiency has also been investigated prospectively, demonstrating the potential of a hypothesis-free approach to identify microbial communities in primary immune deficiencies, solid organ transplantation and hematological diseases (Fourgeaud et al., 2024). In particular, its ability to identify the causal pathogen was found to be more significant in both primary and secondary immune deficiencies than in immunocompetent hosts (Fourgeaud et al., 2024).

Nevertheless, our intention is not to assess mNGS as a diagnostic method per se, as it definitely needs further standardization, appropriate controls for environmental contaminants, and improved scalability. Rather, we aim to highlight the unique opportunity to identify a broader spectrum of microorganisms under previously considered culture-negative conditions while providing additional insight into their metabolic function and pathogenicity. This places mNGS at the forefront of scientific and applied understanding of the role of the microbiome. As highlighted in the aforementioned contributed studies, this approach allows for tailored treatment interventions.

Given that both primary and secondary immune deficiencies can impact the microbiota at different body sites, which, in the context of solid organ transplantation, can further impact end-organ function, we included a review by Elsayed et al. focusing on the urinary microbiome (urobiome) in the context of impaired immune responses. The authors present findings linking the urobiome to different immunologic conditions affecting the transplanted kidney, such as allograft fibrosis. Of particular interest is their discussion on the urinary virome in kidney transplantation. The authors also discuss the implications of the urinary metabolome in immune dysregulation and autoimmunity.

Indeed, the metabolites produced by the constituents of the microbiota are key mediators of local and systemic immune responses in immune deficiency. However, research targeting the complexity of the microbiome-metabolome axis has been relatively limited in immunocompromised hosts, particularly in the context of inborn errors of immunity. Nevertheless, a specific group of short-chain fatty acids (SCFAs) has been of particular interest due to their potential immunoregulatory effects. The review by Jardou et al., which focused on solid organ transplantation, provides insight into the connection between SCFAs, post-transplant dysbiosis, and immunosuppression. The authors indicate the impact of decreased SCFA production under immunosuppressive therapy on low-grade inflammation thus addressing the issue of non-specific allograft injury in relation to microbial shifts. (Jardou et al., 2024).

In summary, at the beginning of this journey, we asked an open question about the opportunities and challenges hidden within the microbiome of immunocompromised hosts. We have only scratched the tip of the iceberg, while the boundary between traditionally perceived “pathogenicity” and “commensalism” remains blurred. This is even more evident in the context of an impaired immune system, where the damage response framework remains highly relevant (Nosanchuk, 2025). However, the ever-evolving and increasingly available multi-omics tools are uncovering opportunities to elucidate the impact of immune deficiency on microbial composition and function identifying biomarkers with diagnostic and prognostic value along with novel therapeutic targets.
